# Carnitine deficiency augments methotrexate-mediated acute renal injury in rats

**DOI:** 10.1186/s43046-025-00321-y

**Published:** 2025-09-26

**Authors:** Fatema Alaa El-Din Selim, Mahmoud M. Khattab, Abdel-Moneim M. Osman, Riham M. Karkeet, Mervat M. Omran, Marwa Sharaky, Mohamed M. Sayed-Ahmed

**Affiliations:** 1 Cairo University, Giza, Egypt; 2 New Giza University, Giza, Egypt

**Keywords:** MTX, L-carnitine, Nephrotoxicity, Mildronate, Carnitine deficiency

## Abstract

**Background:**

This research aimed to explore if carnitine (CARN) deficiency is a risk factor in methotrexate (MTX)-mediated acute kidney injury (AKI) and to mechanistically reveal the potential attenuating effect of CARN against MTX-mediated nephrotoxicity.

**Methods:**

Thirty-six adult male Wister albino rats were subgrouped into six groups. Groups 1, 2, and 3 received 0.9% normal saline (0.5 mL/200 g, i.p.), mildronate (MD, 200 mg/kg/day, i.p.), and l-carnitine (CARN, 200 mg/kg/day, i.p.) for 10 uninterrupted days, respectively. Groups 4, 5, and 6 received similar doses of normal saline, MD, and CARN for 5 days prior to as well as following a solo dose of methotrexate (MTX, 20 mg/kg, i.p.), respectively.

**Results:**

Treatment with a single dose of MTX significantly boosted serum nephrotoxicity as well as hepatotoxicity indices; additionally, it increased the percentage of collagen deposition in rat kidney tissues with obvious histopathological changes. Moreover, MTX lowered kidney levels of adenosine triphosphate (ATP) and amplified acetyl-CoA carboxylase-1 (ACC-1) in kidney tissues. In MD-treated rats, MTX progressively boosted nephrotoxicity indices and collagen disposition in kidney tissues as well as progressive additional reduction in ATP as compared with MTX-treated rats and serum carnitine levels compared with MD-treated rats. Carnitine administration totally counteracted the biochemical and histopathological alterations mediated by MTX to the normal measures.

**Conclusions:**

This research proposes that carnitine deficiency is a potential risk factor in the development of MTX-mediated AKI. MTX disrupts ACC1 signaling with the consequential inhibition of ATP production. Carnitine supplementation attenuates MTX-mediated AKI. Our results are preliminary and mandate further mechanistic study to justify the progression of AKI by MTX in CARN-deficient rats.

## Introduction

Methotrexate (MTX) stands as a unique pharmacological tool with documented success and efficacy in managing various cancers and immune disorders [1, 2]. Its cytotoxic effect is mainly attributed to the blockade of dihydrofolate reductase, thus hindering the formation of activated folic acid [3]. Additionally, MTX opposes thymidylate synthase [4]. MTX has a greatly auspicious cost-effectiveness profile, but unfortunately, perfectionism is unattainable due to its toxicity [5]. The documented incidence of MTX-induced AKI varies between 1.8% and 38.6%, and this can be justified by the non-unified descriptions of acute kidney injury [6–8]. Latcha and colleagues conducted the biggest single-center trial concerned with kidney disease when high-dose MTX is administered after acute kidney injury. They concluded that the acute kidney injury rate was comparable for the overall (32.1%) and survivor patients (37.1%); moreover, acute kidney injury leads to greater mortality [9–11]. As MTX is chiefly excreted by the kidneys, MTX-mediated renal injury leads to delayed clearance of MTX, causing persistent elevation of plasma MTX level, leading to ineffectual rescue by leucovorin and obvious augmentation of MTX’s related toxicities [12]. MTX-related nephrotoxicity has been attributed to oxidative and inflammatory stress, mitochondrial dysfunction, and apoptosis [2, 13].

l-Carnitine (CARN) chief physiological role was discovered in 1962 by Bremer, who announced CARN as a vital cofactor for the transport of long-chain fatty acids from the cytoplasm into the mitochondria, to be utilized for energy production [14]. In 1963, he extended his discovery to contain the carnitine palmitoyl transferase enzyme (CPT I) and documented its pivotal role in long-chain fatty acid oxidation [15]. Previous studies documented that drug-induced nephrotoxicity results in higher excretion of CARN, and its deficiency contributes to as well as exacerbates the drug-related renal insufficiency. Consequently, CARN supplementation amends the severity of adverse effects [16, 17]. CARN supplementation raises cellular carnitine levels and mitochondrial CoA-SH amounts, thus improving mitochondrial oxidative phosphorylation and energy production, and moreover decreases oxidative stress [18]. The cytoprotective effects of CARN are explained by its ability to reduce oxidative stress and maintain the cellular optimum redox status [19]. Moreover, CARN was documented to upturn the genetic expression of peroxisome proliferator-activated receptor-γ coactivator 1-α, a well-established crucial regulator of fatty acid oxidation and the Nrf2 gene, and a famous mitochondrial protector via boosting antioxidant levels and controlling mitophagy [20]. To test the currently suggested hypothesis, mildronate (MD) was utilized to induce carnitine depletion. MD blocks carnitine synthesis, thus avoiding the buildup of toxic by-products resulting from fatty acid beta-oxidation [21]. Mildronate evidenced the ability to genetically control glucose metabolism by altering metabolism from the fatty acids to glucose oxidation, and is reflected as mitochondrial protection from the buildup of acyl-carnitine [20, 22].

There is a critical requisite to find innovative therapeutic interventions for preventing symptoms and adverse events related to MTX [23]. CARN is a well-documented principal cell-protector and should be administered concurrently with various chemotherapeutic medications to hold back their various organ toxic effects and to allow the use of higher doses, resulting in higher cytotoxicity and improved patient survival [24]. Consequently, this research was conducted to explore whether CARN deficiency is a risk factor for MTX-induced acute nephrotoxicity. Furthermore, evaluate whether CARN supplementation can mitigate these detrimental effects, thereby potentially offering a therapeutic strategy to ameliorate methotrexate-induced nephrotoxicity.

## Materials and methods

### Materials

Methotrexate (methotrexate 50 mg/2 mL vials, Mylan S.A.S., France) was provided by the National Cancer Institute, Cairo University, Egypt. l-Carnitine tartarate has been supplied by Dr. Mokhtar Beshr (General Manager and Technical Director of Mepaco Pharmaceuticals, Egypt). Mildronate (Kerui biotechnology Co., Ltd., China) was kindly gifted by Dr. Maha A. Salem, Pharmacology and Toxicology Department, Faculty of Pharmacy, Modern University, for Technology and Information. It has been utilized in the development of carnitine deficiency in rats [25, 26].

### Animals

Adult male Wistar albino rats, with a weight range between 180 and 200 g, were purchased from the Animal Care Center, National Cancer Institute, Cairo University. Rats were contained in cages under controlled environmental conditions (25°C and a 12-h light/dark cycle). Animals have free access to pulverized standard rat pellet diet and water. The protocol of this investigation has been permitted by the Research Ethics Committee (Faculty of Pharmacy, Cairo University) with approval number PT (2963).

## Methods

### Carnitine-depleted mildronate-treated rat model

Carnitine deficiency animal models were established by Paulson and Shug [27], Whitmer [28], and Tsoko et al. [25] Carnitine deficiency was achieved via daily intraperitoneal (I.P.) administration of MD (200 mg/kg/day) for 10 uninterrupted days, following the protocol of Tsoko et al. [25].

### Experimental design

To meet the primary objectives of the research, 36 rats were assigned to six groups and received one of these treatment protocols: Group 1: Normal control rats received 0.9% normal saline (0.5 mL/200 g, i.p) for 10 uninterrupted days. Group 2: Rats received mildronate (200 mg/kg/day, i.p.) for 10 uninterrupted days [25]. Group 3: Rats received l-carnitine (200 mg/kg/day, i.p.) for 10 uninterrupted days [28]. Group 4: Rats received 0.9% normal saline for 5 days prior to and following a single MTX dose (20 mg/kg, i.p) [29, 30]. Group 5: Rats received MD (200 mg/kg/day, i.p.) for 5 days prior to and 5 days following a single MTX dose (20 mg/kg, i.p). Group 6: Rats received l-carnitine (200 mg/kg/day, i.p.) for 5 days prior to and following a solo MTX dose (20 mg/kg, i.p.). On the 11th day, animals were anesthetized with ether, and blood samples were collected by heart puncture followed by serum isolation. Immediately after blood sample withdrawal, animals were sacrificed via decapitation, and the kidneys were then isolated for histopathological and biochemical investigations. A second trial was carried out for the determination of serum MTX level in rats, using a total of 18 rats divided at random into three groups. Group 1: Rats received 0.9% normal saline for 10 uninterrupted days followed by a single MTX dose (20 mg/kg, i.p). Group 2: Rats received MD (200 mg/kg/day, i.p.) for 10 uninterrupted days followed by a solo MTX dose (20 mg/kg, i.p). Group 3: Rats received l-carnitine (200 mg/kg/day, i.p.) for five uninterrupted days, followed by a solo MTX dose (20 mg/kg, i.p.). At 2 h, 4 h, and 24 h following MTX administration, rats were anesthetized using ether, blood samples were collected from the retro-orbital plexus using a sterile hematocrit capillary, and serum was separated for monitoring MTX levels.

### Histopathological examination of kidney tissues

Kidney tissues were used for histopathological studies, as was formerly defined by Culling [31]. In brief, sample fixation was achieved by 10% neutral-buffered formalin, followed by dehydration in serial grades of ethanol, then clearance with xylene and embedment into Paraffin/wax blocks. Five-micron sections were cut by a rotatory microtome and mounted on slides. Tissue sections were stained with hematoxylin and eosin for the determination of histological changes in kidney tissues and Masson’s trichrome stain to discriminate collagen and muscle fibers. Samples were inspected and imaged using a full HD microscopic imaging system (Leica Microsystems GmbH, Wetzlar, Germany). Microscopic scoring was performed by an expert histologist, who was blinded to the treatments. Scores were given as 0= none, 1 = mild, 2 = moderate, and 3 = severe for each of the following criteria: (a) tubular degeneration, (b) interstitial inflammatory cells, and (c) congested glomerular tuft. The microscopic score of the kidney sample was considered as the sum of the scores given to each criterion, and five microscopic fields were analyzed to score each sample. The maximum score recorded was 9 [32, 33].

### Determination of nephrotoxicity indices: blood urea nitrogen and serum creatinine

Blood urea nitrogen (BUN) was determined in accordance with the methods of Tabacco et al. [34], and procedures for measurements were done according to the manufacturer’s instructions (Spectrum Diagnostics, Obour City, Egypt, # 235,001). Creatinine levels were assessed spectrophotometrically using the methods of Fabiny and Ertingshausen [35]. Procedures for measuring serum creatinine were done relying on the manufacturer’s instructions (Spectrum Diagnostics, Obour City, Egypt, # 235,001).

### Determination of hepatotoxicity indices (albumin, aspartate aminotransferase (AST), and alanine aminotransferase (ALT), and bilirubin)

Albumin is measured according to the methods of Doumas et al [36]. The procedure for measurement of serum albumin was carried out relying on the manufacturer’s instructions (Spectrum Diagnostics, Obour City, Egypt, Cat. No. # 211,001). AST was measured based on the methods of Henry et al [37]. The procedure for measurement was done based on the manufacturer’s instructions (Spectrum Diagnostics, Obour City, Egypt, and Cat. No. # 260,001). Procedures for measurement of ALT were done in accordance with the manufacturer’s instructions (Spectrum Diagnostics, Obour City, Egypt, Cat. No. # 264,001). Bilirubin was measured according to the methods of Balistreri and Shaw [38]. Procedures for measurement were done based on the manufacturer’s instructions (Spectrum Diagnostics, Obour City, Egypt, Cat. No. # 225,001).

### Determination of free and total l-carnitine using liquid chromatography-tandem mass spectrometry (LC/MS/MS) analysis

Serum samples used for the determination of carnitine levels were deproteinized by 0.6 M perchloric acid, neutralized to pH 7 using 1.2 M potassium carbonate, and centrifuged at 1000 × g at 4 °C for 5 min. The obtained supernatant has a pH of 7.0 and is now ready for the analysis of free carnitine. To measure the level of total carnitine, the obtained supernatant was mixed with 1 M KOH in an Eppendorf tube and kept at 37 °C for 20 min for the degradation of acylcarnitines. Then, the reaction was stopped with 1 M HCl followed by centrifugation at 1000 × g at 4 °C for 5 min. The resultant supernatant has a pH of 7.0 and is now ready for the analysis of total carnitine. After sample preparation, 250 µL of supernatants containing free and total carnitine were extracted with 750 µL of methanol. The supernatant was relocated to HPLC autosampler vials, and 10 µL was injected onto the LC–MS system. The LC–MS-MS system consisted of an Agilent 1200 HPLC system (Agilent Technologies, CA, USA) coupled to AB SCIEX Q TRAP 3200 mass spectrometer (AB SCIEX, Germany) equipped with an electrospray ionization (ESI) interface. Data acquisition was performed with Analyst 4.0 software (AB SCIEX). The separation was done utilizing Agilent Poroshell 120-C18 (50 mm × 3 mm × 2.7 μm, Agilent). The mobile phase pumped at a flow rate of 300 μL/min consisted of 0.1% heptafluorobutyric acid in both methanol: water (10:90, v/v). Overall run time was 5 min; the method was modified from Sowell et al. [39] Quantification was done with multiple reactions monitoring (MRM) via curtain gas collision-induced dissociation and the following ion transitions: *m*/*z* 162:103 and 162:85 for l-carnitine. Serial dilutions of standards were prepared at concentrations ranging between 88 and 11,300 ng/ml for l-carnitine in drug-free plasma and extracted as described in sample preparation to draw a calibration curve.

### Determination of adenosine triphosphate in kidney tissue

Adenosine triphosphate in renal samples was determined based on the method previously described by Botker et al., [40] utilizing an HPLC system (Jasco Corporation, Ishikawa-Cho, Hachioji, Tokyo, Japan). In short, after deproteinization of the kidney tissue homogenate with ice-cold 6% perchloric acid for 30 min, the pH was neutralized to pH 7.0 using potassium carbonate solution (1.2 M). The supernatant was centrifuged at 3000 rpm for 10 min. At 0.5ºC, the filtered through a 0.2-μm filter and injected into the HPLC system for measurement of ATP. The mobile phase contained ammonium dihydrogen phosphate (75 mM), and the pH was adjusted to 5.7 using ammonia solution, which was then degassed in an ultrasonic bath for 10 min at room temperature. Chromatographic condition was set at a flow rate of 1.2 ml/min, using an ODS-Hypersil column (C18, 25 cm × 4.6 mm) from Supelco SA, Gland, Switzerland. The ATP peaks were detected at 254 nm using a UV detector (Jasco instrument, Tokyo, Japan).

### Determination of acetyl-CoA carboxylase in kidney tissues and serum methotrexate level

The procedure for measuring acetyl-CoA carboxylase was done relying on the manufacturer’s directions using the MyBioSource ELISA kit (Cat. No. # MBS1600641). Assay of serum methotrexate level was carried out according to Borgman and colleagues [41] and Chavan et al. [42] using the Siemens Syva EMIT MTX assay kit.

### Statistical analysis

Differences between measured values (mean ± SD, *n* = 6) were detected by one-way analysis of variance (ANOVA), accompanied by the Tukey–Kramer multiple comparison test aided with GraphPad Prism 5 software. *P* ≤ 0.05 was set as a criterion for a statistical difference.

## Results

### Effect of MTX on histopathological alterations in renal samples in carnitine-depleted and supplemented rats

**Fig. 1:**
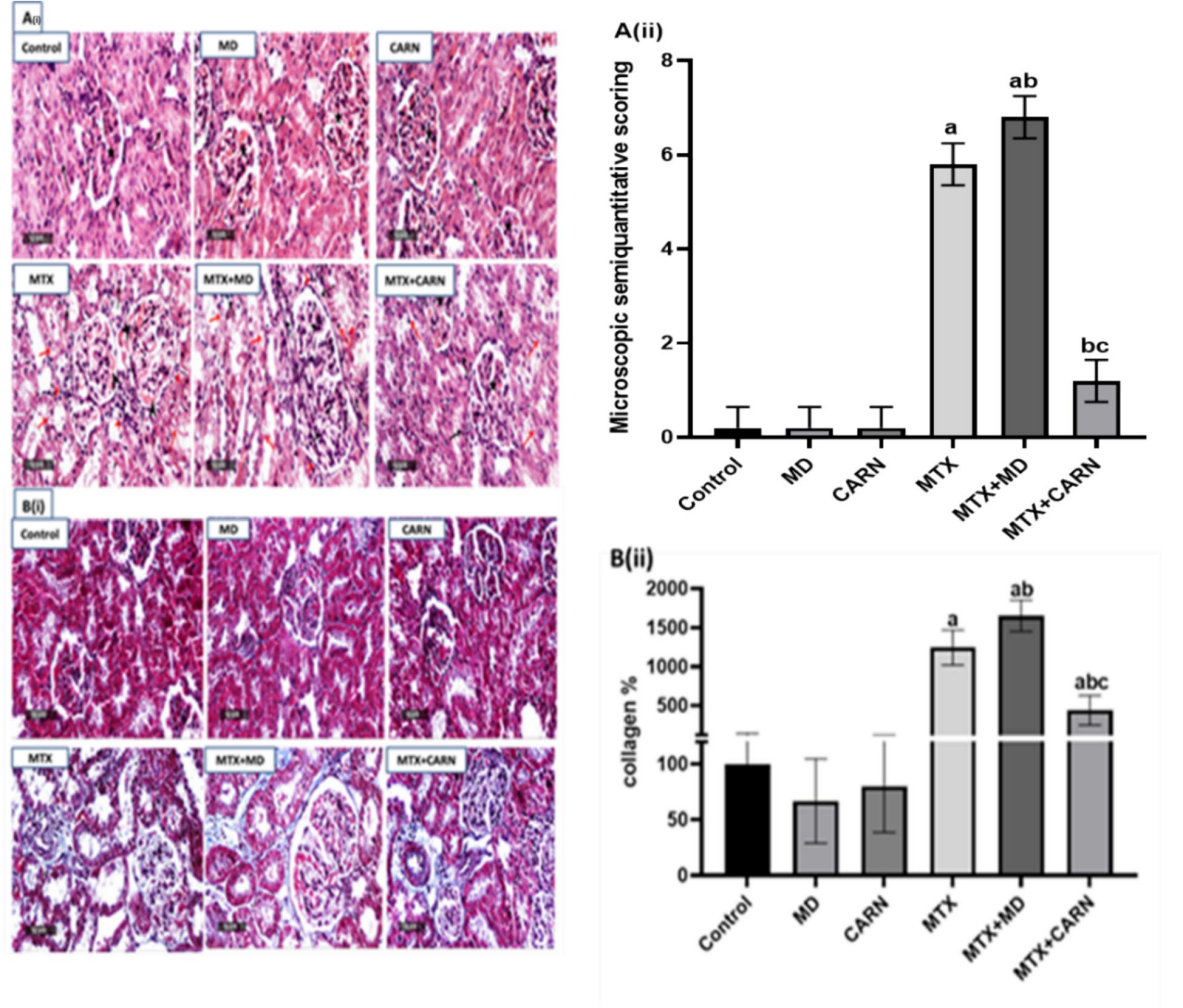
Histopathological alterations in kidney samples of carnitine-depleted andcarnitine-supplemented rats after MTX treatment. Microscopic photomicrographs of renal tissues stained with hematoxylin and eosin. Figure 1Ai shows the microscopic photographs, and 1Aii shows a semi-quantitative bar chart for the histopathological alterations. Masson’s trichrome (**1Bi**) and quantitative bar chart of percentage of collagen deposition (**1Bii**). Results are depicted as mean ± SD (*n* = 6). Panels a, b, and c show significant change from control, MTX, and MTX-MD, respectively, at *p* < 0.05 using one-way ANOVA accompanied by Tukey–Kramer as a post-ANOVA test. MD, mildronate; CARN, l-carnitine; MTX, methotrexate

Figure 1 shows the histopathological alterations in kidney samples stained with (A) hematoxylin and eosin (HE, X200); Fig. 1Ai shows the microscopic photographs, and 1Aii shows a semi-quantitative bar chart for the histopathological alterations and (B) Masson’s trichrome (MT, X200). Figure 1A demonstrated that normal control samples (group 1) showed normal histological structures of renal parenchyma with obvious integral kidney corpuscles (star), renal tubular segments with nearly integral tubular epithelium (arrow), and integral vasculatures. Also, both mildronate samples (group 2) and carnitine samples (group 3) showed almost intact morphological features without abnormal microscopic changes (as normal control). Moreover, MTX-treated animals (group 4) demonstrated moderate records of tubular degeneration with significant loss of luminal border integrity of proximal and distal convoluted tubules (red arrows) with occasional luminal detached epithelial cells (black arrow), marked congested and dilatation of glomerular tuft capillaries (black arrow head), mild periglomerular and interstitial mononuclear inflammatory cells infiltrates were observed (red arrow head) accompanied with mild interstitial and perivascular fibrosis in Masson’s trichrome-stained sections. Interestingly, MTX + mildronate samples (group 5) showed more severe tubular damage and degenerative changes (red arrow) accompanied with hyperplasia of mesangial cells of renal corpuscles (black arrow head), mild interstitial inflammatory cells infiltrates were showed (red arrow head) with obvious higher fibroblastic activity as well as periglomerular and interstitial fibrosis (dashed arrow). On the contrary, MTX + carnitine samples (group 6) showed significant protective efficacy on renal parenchyma with almost intact kidney corpuscles (star), mild focal records of tubular epithelial damage (red arrow) alternated with obvious integral tubular segments (black arrow) and minimal interstitial infiltrates. However, scanty few records of periglomerular and perivascular collagen fibers could be observed in Masson’s trichrome-stained tissue sections.

Figure 1B shows the microscopic photograph (1Bi) and quantitative bar chart (1Bii) for the effects of MTX on the percent of collagen deposition in carnitine-supplemented and depleted rats. Administration of a solo injection of MTX induced an 11.44-fold elevation in collagen deposition compared with the control group. Daily injection of MD for 5 days before and after a solo dose of MTX resulted in a 15.5-fold and fourfold rise in BUN as compared with the control and MTX groups, respectively. Daily injection of CARN for 5 days before and after a single dose of MTX induced an eightfold and 12-fold reduction of collagen deposition compared with the MTX and MTX-MD groups, respectively. Challenging with either CARN or MD alone for 10 uninterrupted days showed non-significant alterations in collagen deposition.

### Effect of MTX on blood urea nitrogen and serum creatinine levels in carnitine-depleted and supplemented rats

**Fig. 2:**
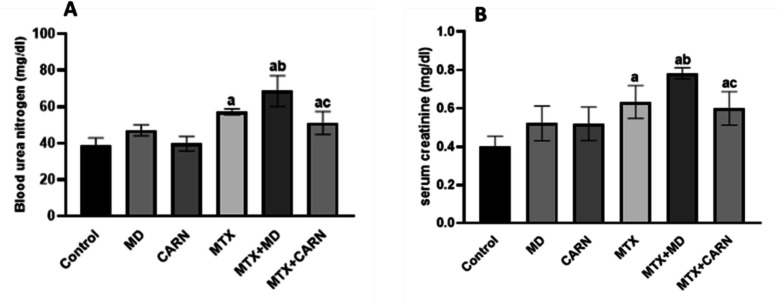
Effect of MTX on BUN (**A**) and serum creatinine (**B**) levels in carnitine-depleted and supplemented rats. Results are depicted as mean ± SD (*n* = 6). Panels a, b, and c show significant change from control, MTX, and MTX-MD, respectively, at *p* < 0.05 using one-way ANOVA accompanied by Tukey–Kramer as a post-ANOVA test

Figure 2 demonstrates the impact of MTX on BUN (A) and serum creatinine (B) in carnitine-supplemented and depleted rats. Challenging with a solo dose of MTX induced 47% and 58% boost in BUN and creatinine, respectively, compared with the control. Daily challenge with mildronate (MD) for 5 successive days before and after a solo dose of MTX acheived 76% and 20% elevation of BUN as well as 96% and 23% elevation in serum creatinine compared with control and MTX groups, respectively. Daily challenge with l-carnitine (CARN) for 5 days before and after a solo dose of MTX caused 11% and 25% reduction in BUN compared with the MTX and MTX-MD groups, respectively; moreover, a 23% decline in serum creatinine level compared with the MTX-MD group. Challenging with either CARN or MD alone for 10 uninterrupted days showed nonsignificant fluctuations in BUN or creatinine compared to control.

#### Effect of MTX on the albumin in carnitine-depleted and supplemented rats

**Fig. 3:**
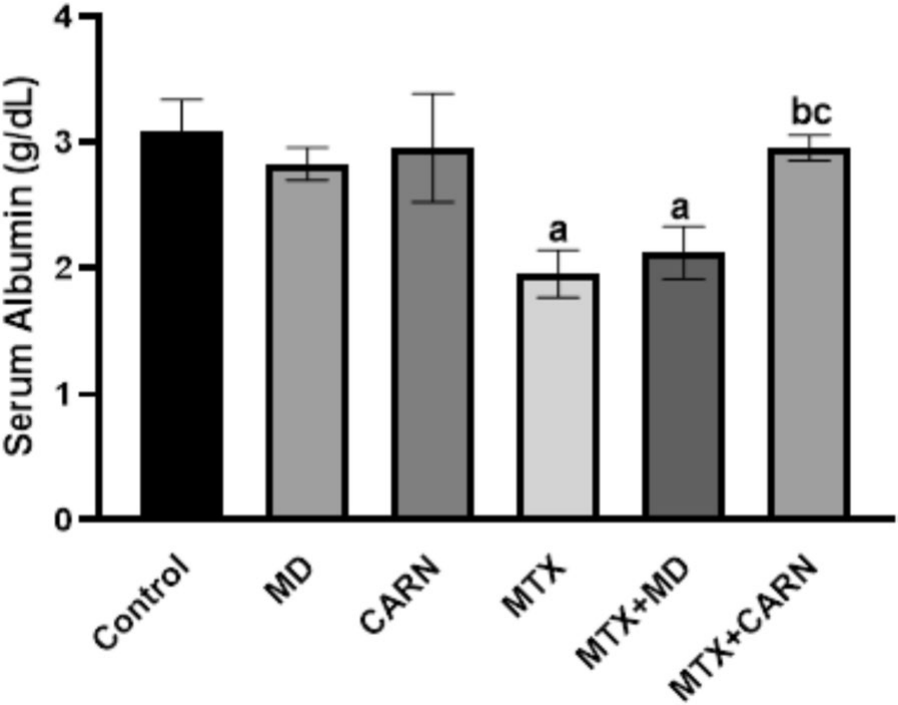
Effect of MTX on serum albumin level in carnitine-depleted and supplemented rats. Results are depicted as mean ± SD (*n* = 6). Panels a, b, and c show significant change from control, MTX, and MTX-MD, respectively, at *p* < 0.05 using one-way ANOVA accompanied by Tukey–Kramer as a post-ANOVA test

The influence of MTX on serum albumin level in carnitine-depleted and supplemented rats is demonstrated in Fig. 3. Treatment with MTX (20 mg/kg) caused a 36% reduction in serum albumin compared to the control. Injecting MTX into carnitine-depleted rats caused a 31% reduction in serum albumin compared with the control. Daily challenge with CARN for 5 days before and after a single dose of MTX induced 51% and 27% increase in serum albumin level compared with the MTX and MTX-MD groups, respectively. Challenging with either CARN or MD alone for 10 uninterrupted days showed non-significant alterations in serum albumin compared with control.

### Effect of MTX on ALT and AST in carnitine-depleted and supplemented rats

**Fig. 4:**
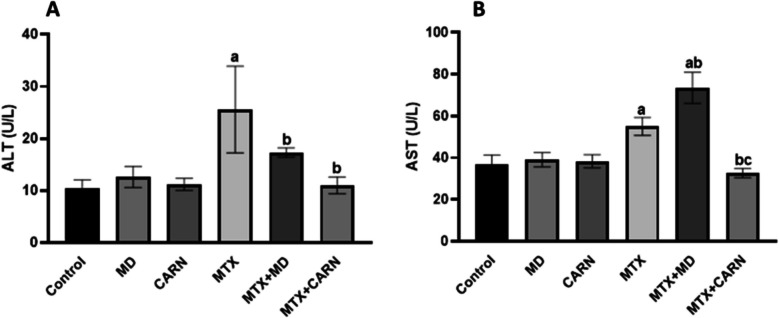
Effect of MTX on serum ALT (**A**) and AST (**B**) levels in carnitine-depleted and supplemented rats. Results are depicted as mean ± SD (*n* = 6). Panels a, b, and c show significant change from control, MTX, and MTX-MD, respectively, at *p* < 0.05 using one-way ANOVA accompanied by Tukey–Kramer as a post-ANOVA test

The impacts of MTX on serum ALT and AST levels in carnitine-depleted and supplemented rats are illustrated in Fig. 4. Treatment with MTX (20 mg/kg) caused 145% and 49% elevation in serum ALT (A) and AST (B), respectively, compared with the control. Injecting MTX into carnitine-depleted rats achieved 66% and 99.5% elevation in serum ALT and AST, respectively, compared with the control, and 34% elevation compared with the MTX group. Daily challenge with CARN for 5 days before and after a solo dose of MTX caused 60% and 111% reduction in serum ALT and AST levels, respectively, compared with MTX-MD. Challenging with either CARN or MD alone for 10 uninterrupted days showed a non-significant change.

### Effect of MTX on bilirubin level in carnitine-depleted and supplemented rats

**Fig. 5:**
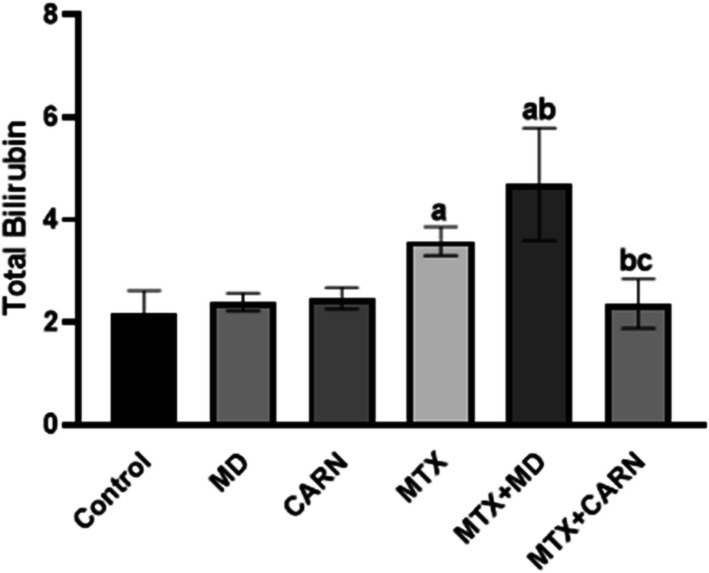
Effect of MTX on serum bilirubin level in carnitine-depleted and supplemented rats. Results are depicted as mean ± SD (*n* = 6). Panels a, b, and c show significant change from control, MTX, and MTX-MD, respectively, at *p* < 0.05 using one-way ANOVA accompanied by Tukey–Kramer as a post-ANOVA test

The impacts of MTX on serum bilirubin level in carnitine-depleted and supplemented rats are presented in Fig. 5. Treatment with MTX (20 mg/kg) caused a 65% boost in serum bilirubin compared with the control. Challenging with MTX to carnitine-depleted rats achieved 116% and 31% elevation in serum bilirubin compared with the control and MTX groups, respectively. Daily injection of CARN for 5 days before and after a solo dose of MTX caused a 107% reduction in serum bilirubin level compared with MTX-MD. Challenging with either CARN or MD alone for 10 uninterrupted days showed non-significant changes in serum bilirubin compared to control.

### Effect of MTX on serum total and free carnitine levels in carnitine-depleted and supplemented rats

**Fig. 6:**
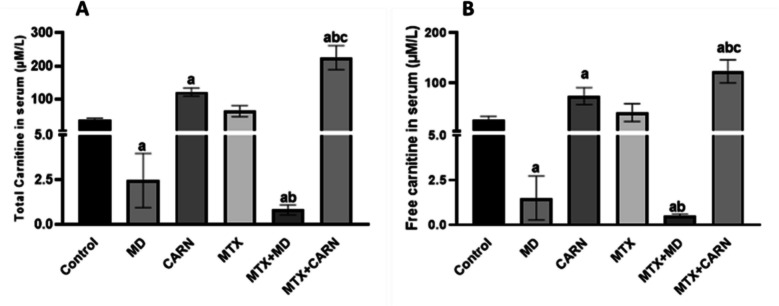
Effect of MTX on serum total (**A**) and free (**B**) carnitine levels in carnitine-depleted and supplemented rats. Results are depicted as mean ± SD (*n* = 6). Panels a, b, and c show significant change from control, MTX, and MTX-MD, respectively, at *p* < 0.05 using one-way ANOVA accompanied by Tukey–Kramer as a post-ANOVA test

The impacts of MTX on serum total and free carnitine concentrations in carnitine-depleted and supplemented rats are presented in Fig. 6. Injecting MD alone for 10 uninterrupted days showed a 94% decrease in total (A) and free (B) carnitine compared with the control. Injecting a solo dose of MTX (20 mg/kg) caused a non-significant 66% and 54% elevation in total and free carnitine, respectively, compared with the control. Oppositely, injecting CARN alone for 10 uninterrupted days caused a noticeable elevation in serum total and free carnitine by twofold and 1.8-fold, respectively, compared with the control group. Injecting MTX to carnitine-depleted rats caused 98% and 67% reduction in serum total carnitine and 98% and 65% reduction in serum free carnitine compared to control and MD groups, respectively. Daily treatment with CARN for 5 days before and after a single dose of MTX achieved 4.8-fold and 26.5-fold elevation in serum total carnitine and 3.57-fold and 22.6-fold elevation in serum-free carnitine level compared with control and MTX-MD groups, respectively.

### Time-course for serum MTX level in carnitine-depleted and supplemented rats

**Fig. 7:**
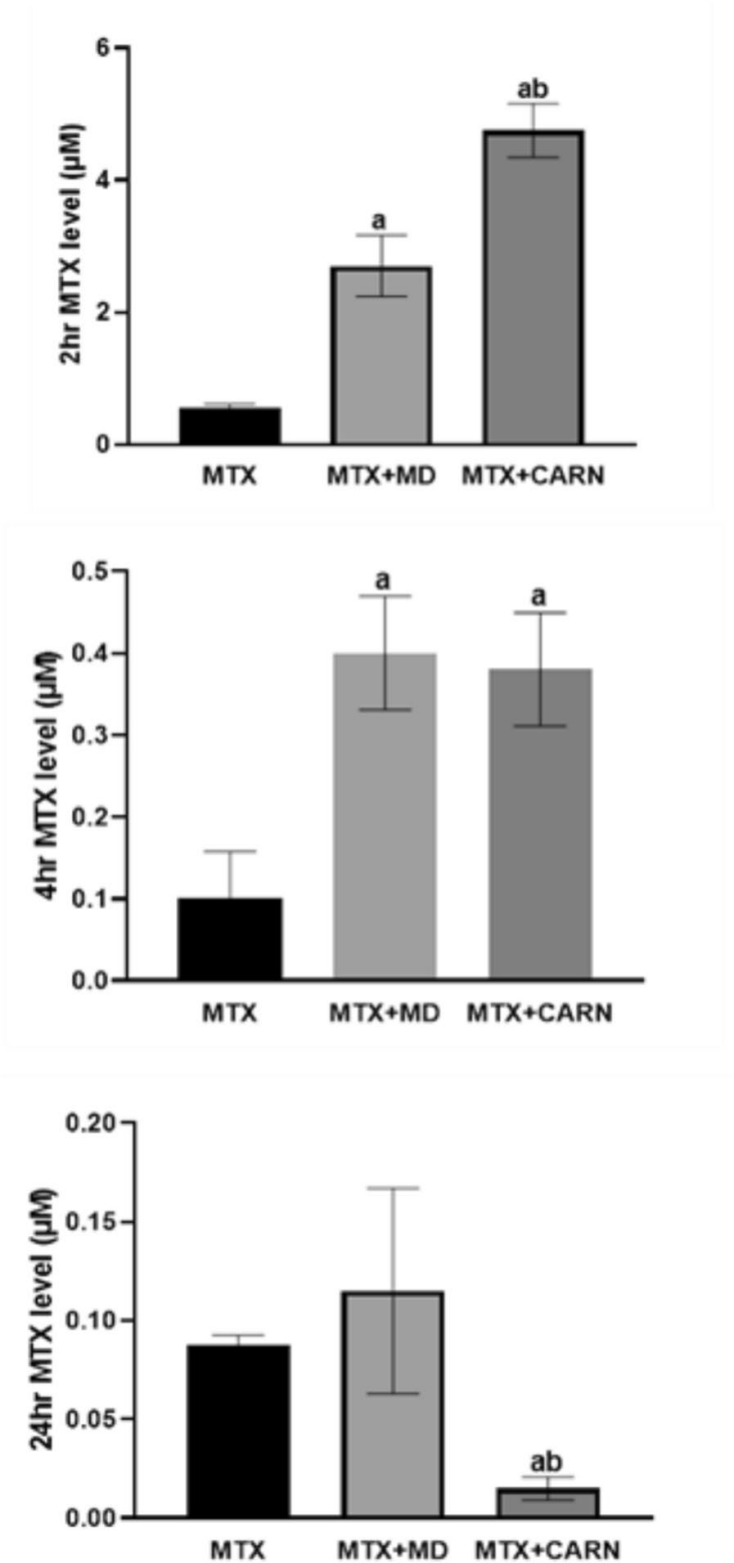
Time-course for serum MTX level in carnitine-depleted and supplemented rats. Results are depicted as mean ± SD (*n* = 6). Panels a and b show significant change from MTX and MTX-MD, respectively, at *p* < 0.05 using one-way ANOVA accompanied by Tukey–Kramer as a post-ANOVA test

The effects of carnitine depletion and supplementation on serum MTX levels are shown in Fig. 7. At 2 and 4 h after MTX administration, serum MTX levels were elevated in both carnitine-depleted and supplemented rats compared with MTX alone. However, at 24 h, carnitine supplementation significantly decreased MTX levels as compared with the MTX and MTX + MD groups.

### Effect of MTX on the ACC1 in carnitine-depleted and supplemented rats

**Fig. 8:**
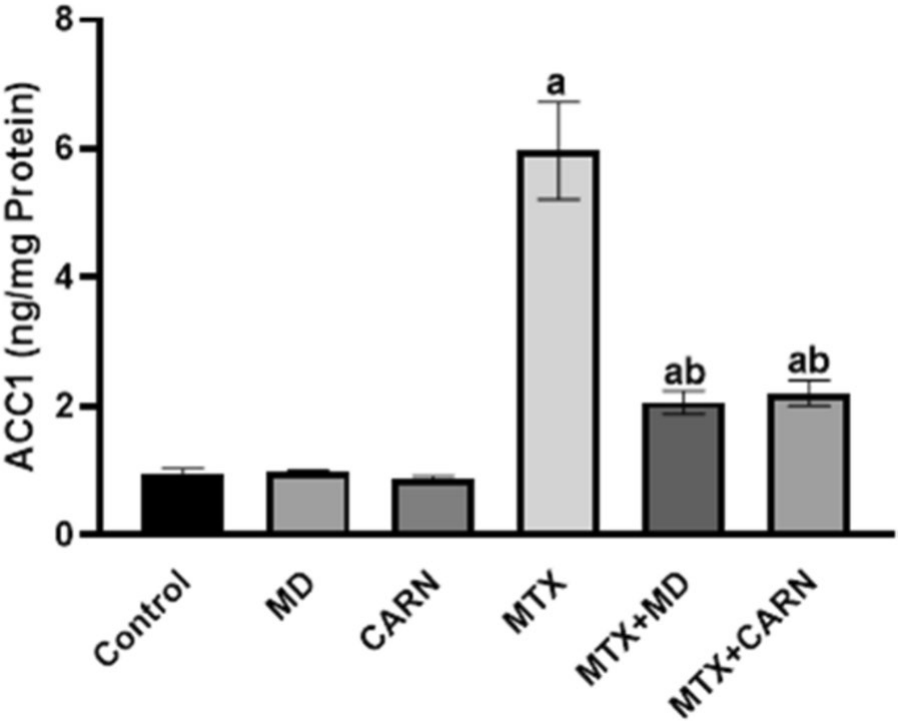
Effect of MTX on ACC1 in carnitine-depleted and supplemented rats. Results are depicted as mean ± SD (*n* = 6). Panels a, b, and c show significant change from control, MTX, and MTX-MD, respectively, at *p* < 0.05 using one-way ANOVA accompanied by Tukey–Kramer as a post-ANOVA test

The impacts of MTX on ACC1 in carnitine-depleted and supplemented rats are presented in Fig. 8. Treatment with MTX (20 mg/kg) caused a 5.2-fold elevation in ACC1 compared with control. Challenging with MTX to carnitine-depleted rats achieved 113% elevation and 65% reduction in ACC1 compared with control and MTX groups, respectively. Daily challenge with CARN for 5 days before and after a solo dose of MTX showed 128% elevation and 63% reduction in ACC1 compared with the control and MTX groups, respectively. Challenging with either MD or CARN alone for 10 uninterrupted days showed a non-significant change.

### Effect of MTX on kidney ATP in carnitine-depleted and supplemented rats

**Fig. 9:**
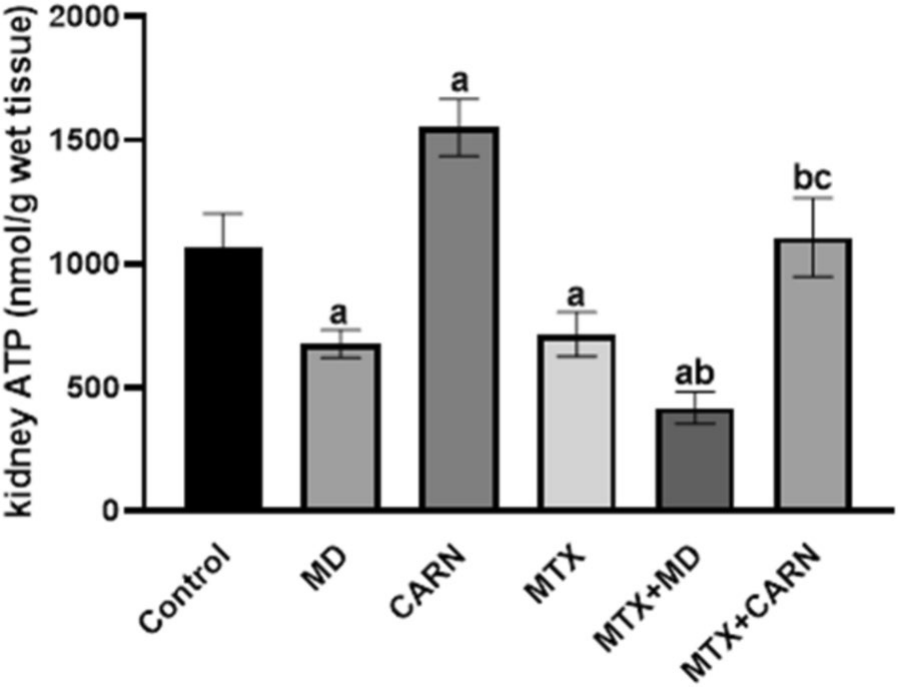
Effect of MTX on kidney ATP in carnitine-depleted and supplemented rats. Results are depicted as mean ± SD (*n* = 6). Panels a, b, and c show significant change from control, MTX, and MTX-MD, respectively, at *p* < 0.05 using one-way ANOVA accompanied by Tukey–Kramer as a post-ANOVA test

The effects of MTX on kidney ATP in carnitine-depleted and supplemented rats are presented in Fig. 9. Treatment with MTX (20 mg/kg) caused a 33% reduction in renal ATP compared with control. Challenging with MTX to carnitine-depleted rats achieved more profound 61% and 42% reduction in kidney ATP compared with control and MTX groups, respectively. Daily challenge with CARN for 5 days before and after a solo dose of MTX caused normalization of MTX-induced reduction in ATP. Injecting MD alone for 10 uninterrupted days showed a 37% reduction in kidney ATP compared with the control group. Challenging with CARN alone for 10 uninterrupted days achieved a 46% elevation in kidney ATP compared with the control group.

## Discussion

Methotrexate (MTX) is an everlasting medication used in a wide range of cancerous and benign maladies [43]. It stands as an extraordinary candidate for drug repositioning; originally developed as an anti-cancer agent [43], then extending to numerous autoimmune diseases [44], and currently repurposed antimicrobial medication [45]. Methotrexate-associated nephrotoxicity is a serious and mostly inevitable side effect that can be due to an allergic reaction or direct drug toxicity to the renal tubules [46]. Thus, therapeutic monitoring of methotrexate, encouraging its excretion and pharmacological opposition of its nephrotoxicity, is of great importance [47, 48]. The ideal clinical efficacy of chemotherapy is unachievable owing to its deleterious toxicities, and extensive expert research has documented that these toxicities could mainly be attributed to secondary carnitine insufficiency [49]. Moreover, carnitine deficiency has been proven as a contributing factor to various chemotherapeutic drug-induced toxicities; thus, its replacement attenuates and reverses the medication-induced adverse effects without compromising the anti-tumor activity [17]. Accordingly, the current study aimed to explore if carnitine deficiency is a surplus aggravating factor and/or a mechanism in methotrexate-mediated acute nephrotoxicity and to evaluate the probable mechanistic protective influence of L-carnitine against nephrotoxicity.

The current data revealed that a single dose of MTX triggered AKI on histopathological and biochemical levels. MTX caused moderate tubular degeneration as well as loss of luminal border integrity of proximal and distal convoluted tubules, obvious congestion and dilatation of glomerular tuft capillaries, and other pathological changes. The methotrexate-induced degenerative changes were more profound in carnitine carnitine-deficient group. Conversely, the carnitine-supplemented group showed a significant renoprotective effect to restore the field appearance to the control samples. The changes were also observed in collagen deposition percentage, where methotrexate alone boosted it and more profound deposition was observed in carnitine-deficient MD-treated rats. Contrariwise, carnitine supplementation lessened the MTX-induced renal collagen deposition. Under the current experimental conditions, the observed histopathological damages by MTX in kidney tissues confirm those previously reported [29, 30]. Moreover, MTX-mediated AKI was confirmed by elevated serum creatinine and blood urea nitrogen (BUN). In contrast, challenging with MTX to carnitine-depleted MD-treated rats aggravated nephrotoxicity. The presently recorded deterioration in kidney performance markers could be attributed to the metabolic drifting to carnitine-free or protein energy sources. This assumption could be allied with the results documented by Ahmed et al., [50] which showed that carnitine supplementation along with hemodialysis boosted protein anabolism, this was reflected by diminished protein metabolic byproducts as BUN and creatinine [50]. The progression of chemotherapy-induced AKI under the condition of carnitine deficiency has been previously reported [17, 51]. Recently, Ulinski et al. reviewed the role of carnitine supplementation in disease-associated and drug-induced AKI in clinical practice [52]. Radwan et al. documented the defensive role of carnitine against methotrexate nephrotoxicity by refining the histopathological appearance, reducing the oxidative stress as well as the inflammatory cascade and programmed cell death. Also, carnitine was proven to augment the expression of renal SIRT1/PGC-1/Nrf2/HO-1 in a rodent model of MTX-mediated nephrotoxicity [53]. Sener et al. proved the oxidative stress implicated by methotrexate and attributed it to augmenting the MDA and MPO, increasing collagen deposition, and reducing GSH; moreover, it increased the level of TNF-alpha and leukocyte apoptosis. Furthermore, degenerative changes of multiple organs, including the liver and glomerular and tubular epithelium, were observed. Methotrexate-induced changes were reversed by carnitine replacement [54]. In harmony, the current findings showed improved histomorphological appearance and kidney functions upon restoring carnitine levels.

In the current study, methotrexate-induced hepatotoxicity was documented by increased AST, ALT, and bilirubin levels compared with the control. Hepatotoxicity indices were measured because, beside the kidneys, the liver is the chief organ in charge of carnitine production, justifying the rationale behind assessing both organs’ performance. The last product in carnitine biosynthesis, butyrobetaine, is released into the bloodstream and reuptaken by the liver and kidney, then hydroxylated on the third carbon by butyrobetaine hydroxylase to form L-carnitine [55]. The methotrexate liver toxicity was more aggressive when combined with mildronate and reversed to normal control levels upon supplementation with carnitine, emphasizing its magical protective effect. Carnitine’s impact on liver performance was extrapolated with albumin, where methotrexate reduced it, with a more profound reduction in carnitine-deficient model and reversion to normal levels when combined with carnitine. A previous study pointed to the modulatory effect of carnitine on the Notch1/Hes-1 signaling pathway. It proved that carnitine can counteract the methotrexate boosting effect on Notch1 and Hes-1 proteins and level-down TNF-α, interleukin (IL)−6, and IL-1β in the liver; thus relieving methotrexate liver toxicity [56]. Golpayegani et al. revealed that oral carnitine supplementation with methotrexate improves liver enzymes with minimal carnitine-induced side effects [57]. Detailed description of methotrexate imposed liver cell changes was documented by Kadry et al., where all cellular organelles showed dramatic irregular changes, including mitochondria. Concurrent carnitine supplementation has been proven to ameliorate methotrexate hepatotoxicity more than sequential administration [58].

It is well established that mildronate inhibits gamma-butyrobetaine hydroxylase, which is crucial for carnitine biogenesis, thus offering an experimental rodent model for carnitine deficiency [59]. Although MTX caused an insignificant rise in CARN compared with control, in carnitine-depleted MD-treated rats, MTX caused a progressive decrease in serum total and free carnitine. The observed progressive increase in nephrotoxicity and hepatoxicity indices in carnitine-depleted rats was aligned with the progressive decrease in serum carnitine, which may justify the possible consideration of carnitine deficiency as a risk factor in MTX-mediated acute nephrotoxicity and hepatotoxicity. Surprisingly, the MTX combination with carnitine boosted its level to a higher extent, even compared with CARN alone. Logically, these results were mirrored in the level of free carnitine. One of the most detrimental metabolic changes in cancer patients is reduced synthesis of carnitine, which is aggravated by the lowered dietary intake [60]. It is well documented that carnitine assists the β-oxidation process, enhances energy metabolism, counteracts oxidative stress, and decreases apoptosis [61, 62]. To evaluate the impact of carnitine on energy metabolism, the kidney ATP level was assessed. It revealed the enhancing effect of carnitine as well as the detrimental effects of both mildronate and methotrexate. Methotrexate’s negative impact was more pronounced in carnitine-deficient model, but fortunately, carnitine supplementation succeeded in reversing the kidney ATP level to the control level. It is well known that L-carnitine is a crucial player in the transportation of free fatty acids to the mitochondria for beta-oxidation and production of acetyl-CoA [63]. Acetyl CoA is a fundamental precursor for ATP production, which is essential for counteracting oxidative stress [64]. These facts can explain the current observations regarding kidney ATP level and confirm the antioxidant potential of carnitine. For further investigation of carnitine potential in reversing MTX impact on energy status, the current work extended to assessing acetyl-CoA carboxylase (ACC1), which is an established controller of energy homeostasis [65]. AMP-activated protein kinase (AMPK) blocks fatty acid production and stimulates fatty acid oxidation by phosphorylation of acetyl-CoA carboxylase ACC1 [66] Activated AMPK inhibits ACC activity via phosphorylation [67]. Phosphorylation of ACC1 is documented as essentially related to AMPK physiology and considered a surrogate indicator of AMPK stimulation. Thus, ACC1 phosphorylation is crucial to AMPK signaling [68]. ACC1 is the physiological converter of acetyl-CoA to malonyl-CoA, and the latter is the Spark for fatty acid production, as well as a blocker to carnitine palmitoyl transferase 1 (CPT1) [67, 69]. Consequently, activated ACC results in the elevation of malonyl-CoA and reduced function of CPT1 and thus increased production of fatty acid [70]. Concisely, reduced cellular ATP activates AMPK, which inhibits ACC1 via phosphorylation, leading to increased fatty acid oxidation and restoring energy stores [71]. In the current context, MTX (20 mg/kg) achieved a 33% reduction in kidney ATP and a five-fold shooting in ACC1. MTX in carnitine-depleted caused a more profound 61% reduction in kidney ATP and 114% increase in ACC1. CARN administration for 10 uninterrupted days showed an elevation in kidney ATP by 46%, whereas MD alone for 10 uninterrupted days showed a significant 37% reduction in kidney ATP, but no significance was reached for ACC1. Thus, carnitine replacement can counteract MTX-induced ATP reduction via modulation of fatty acid oxidation.

Physiologically, L-carnitine is efficiently preserved by its reabsorption to almost 95% via an explicit, high-affinity, sodium-dependent transporter named OCTN2 [72]. It was documented that lower organ carnitine after chemotherapeutic agents is attributed to the restrained production and/or limited reabsorption. Moreover, DNA-associated chemotherapy evidently inhibited OCTN2 mRNA and protein expression [73]. MTX clearance is PH dependent, and this lifesaving fact is used to treat its increased plasma concentrations via urine alkalinization [74]. MTX PH dependency explains its interaction with numerous medications as NSAIDs, proton pump inhibitors, and probenecid [75]. Herein, the current Study used carnitine tartarate for 10 days with consequent lowering of urine PH to around 4, which might explain the increased level of MTX due to reduced clearance [76]. MTX is chiefly eliminated by the kidneys through active secretion and undergoes significant enterohepatic recirculation [77, 78]. In this context, to explain the current results from a pharmacokinetics perspective, the observed differences in serum MTX levels among the three groups of rats can be attributed to pharmacokinetic alterations influenced by carnitine status. Recorded levels of MTX after 24 h of administration showed that in CARN-depleted rats, the observed higher MTX level could be due to lowered excretion, as CARN has been proven to impact energy metabolism in renal tubules [79]. Thus, CARN depletion may impair active tubular secretion of MTX, leading to decreased clearance and higher serum levels. Moreover, CARN deficiency may alter distribution, hindering MTX cellular uptake into tissues, resulting in higher serum levels [80]. Furthermore, CARN depletion could lead to the accumulation of endogenous substrates as acyl-carnitines that compete with MTX for transporters, delaying its elimination by competitive inhibition [81]. On the other hand, CARN-supplemented rats showed reduced MTX levels, which may be explained by enhanced excretion due to improved renal tubular function [79]. Also, CARN may have enhanced MTX distribution into cells, reducing its serum levels. Concisely, CARN status was speculated to affect MTX clearance, volume of distribution, and transporter interactions. This study suggests carnitine’s role in modulating MTX pharmacokinetics, particularly in excretion and/or tissue partitioning. The authors recommend conducting further studies on transporter dynamics and carnitine-MTX interactions to clarify the exact mechanisms. In conclusion, the current findings prove that carnitine replacement to restore its homeostasis is a golden strategy to prevent as well as attenuate methotrexate induced organ toxicity via reversing its impact on ACC1, ATP levels, and kidney and liver functions. The current results suggest that carnitine levels might be used as indicators for toxicity and a monitoring aid during methotrexate therapy. Moreover, carnitine deficiency aggravates methotrexate-related toxicities. Accordingly, authors highly recommend carnitine supplementation with methotrexate therapy to attenuate or prevent the possible acute kidney injury adverse effect. To strengthen the study’s validity, it would be beneficial to assess factors such as CPT I, the key enzyme controlling the mitochondrial transportation of long-chain fatty acids, OCTN2, the carnitine transporter gene, AMPK, and malonyl-CoA. Moreover, the impacts of carnitine supplementation and depletion on the MTX antitumor effects are not explored. Therefore, the preliminary findings from this study warrant the need for a comprehensive mechanistic investigation to examine the effect of AMPK downregulation and MTX kinetics in a murine tumor-bearing model under conditions of carnitine supplementation and depletion to further investigate their impact on the antitumor effect of MTX and MTX-induced kidney injury. In conclusion, this study indicates that carnitine deficiency may contribute to the onset of MTX-induced AKI. MTX disrupts ACC1 signaling, resulting in decreased ATP production in the kidney. Contrariwise, carnitine supplementation appears to alleviate the damage caused by MTX-induced acute kidney injury.

## Data Availability

No datasets were generated or analysed during the current study.

## Abbreviations

CARN: L- carnitine
MD: Mildronate
MTX: Methotrexate
AMPKα1: Adenosine monophosphate-activated protein kinase
ACC-1: Acetyl-CoA carboxylase-1
ATP: Adenosine triphosphate
BUN: Blood urea nitrogen
AST: Aspartate aminotransferase
ALT: Alanine aminotransferase
